# Tumor-derived small extracellular vesicles: potential roles and mechanism in glioma

**DOI:** 10.1186/s12951-022-01584-6

**Published:** 2022-08-23

**Authors:** Xu Guo, Rui Sui, Haozhe Piao

**Affiliations:** 1 Department of Neurosurgery, Cancer Hospital of China Medical University, Liaoning Cancer Hospital & Institute, No. 44 Xiaoheyan Road, Shenyang, 110042 Liaoning China; 2 Department of Neurosurgery, Cancer Hospital of Dalian University of Technology (Liaoning Cancer Hospital & Institute), No. 44 Xiaoheyan Road, Shenyang, 110042 Liaoning China

**Keywords:** Glioma, Tumor-derived SEVs, Malignant progression, Immunotherapies

## Abstract

Small extracellular vesicles (SEVs) are extracellular vesicles containing DNA, RNA, and proteins and are involved in intercellular communication and function, playing an essential role in the growth and metastasis of tumors. SEVs are present in various body fluids and can be isolated and extracted from blood, urine, and cerebrospinal fluid. Under both physiological and pathological conditions, SEVs can be released by some cells, such as immune, stem, and tumor cells, in a cytosolic manner. SEVs secreted by tumor cells are called tumor-derived exosomes (TEXs) because of their origin in the corresponding parent cells. Glioma is the most common intracranial tumor, accounting for approximately half of the primary intracranial tumors, and is characterized by insidious onset, high morbidity, and high mortality rate. Complete removal of tumor tissues by surgery is difficult. Chemotherapy can improve the survival quality of patients to a certain extent; however, gliomas are prone to chemoresistance, which seriously affects the prognosis of patients. In recent years, TEXs have played a vital role in the occurrence, development, associated immune response, chemotherapy resistance, radiation therapy resistance, and metastasis of glioma. This article reviews the role of TEXs in glioma progression, drug resistance, and clinical diagnosis.

## Introduction

Glioma is one of the most common primary malignant tumors in the adult central nervous system, accounting for approximately 70% of all primary intracranial tumors. More than half of them are the most malignant-glioblastoma multiforme (GBM) [1]. Although surgical treatment, radiotherapy, chemotherapy, and gene and immunotherapy have been significantly developed for gliomas in recent years, satisfactory results have not been achieved in clinical practice. Gliomas still have low cure rates, high recurrence rates, and poor prognosis [2–4]. The immunosuppressive tumor microenvironment that develops during glioma and infiltrative growth of glioma are the main reasons for the poor outcome of glioma treatment [5–8].

Small extracellular vesicles (SEVs) are a class of secretory vesicles with a single membrane structure in the form of cups or spheres, with a particle size of approximately 30–150 nm. They are rich in proteins, lipids, nucleic acids, and other biological information materials, and they can serve as a carrier of intercellular information transfer [9]. SEVs are present in almost all body fluids, such as blood and sweat, and almost all body cells can secrete SEVs [10, 11]. SEVs can be involved in various biological processes in a living organism, help in information transfer between cells, and play a vital role in the cellular microenvironment [12–15]. In the tumor microenvironment (TME), SEVs have an essential information transfer function, enabling the transmission of information between tumor cells. SEVs can effectively carry and deliver molecular signals to recipient cells, promoting various biological processes such as tumor growth, metastasis, invasion, tumor angiogenesis, tumor innervation, and chemotherapeutic drug resistance [16–22]. SEVs have been found in many types of cells, and tumor cell-derived exosomes (TEXs) are becoming a hot topic in oncology research due to their properties and functional characteristics [23–25]. TEXs can release their cytokines to promote tumorigenesis, development, proliferation, and migration and make tumors resistant to drugs, adversely affecting tumours’ treatment [26–28].

The TME is a local homeostatic environment composed of tumor cells, macrophages, fibroblasts, and extracellular matrix (ECM), which plays a significant role in cancer initiation, metastasis, recurrence, and chemotherapy resistance [29–31]. This review focuses on the biological properties of SEVs and the role and molecular mechanisms of TEXs in glioma development and progression and intends to provide novel insights into the developments in the clinical diagnosis and treatment of glioma.

## Biogenesis, contents, and main biological function of SEVs

**Fig. 1:**
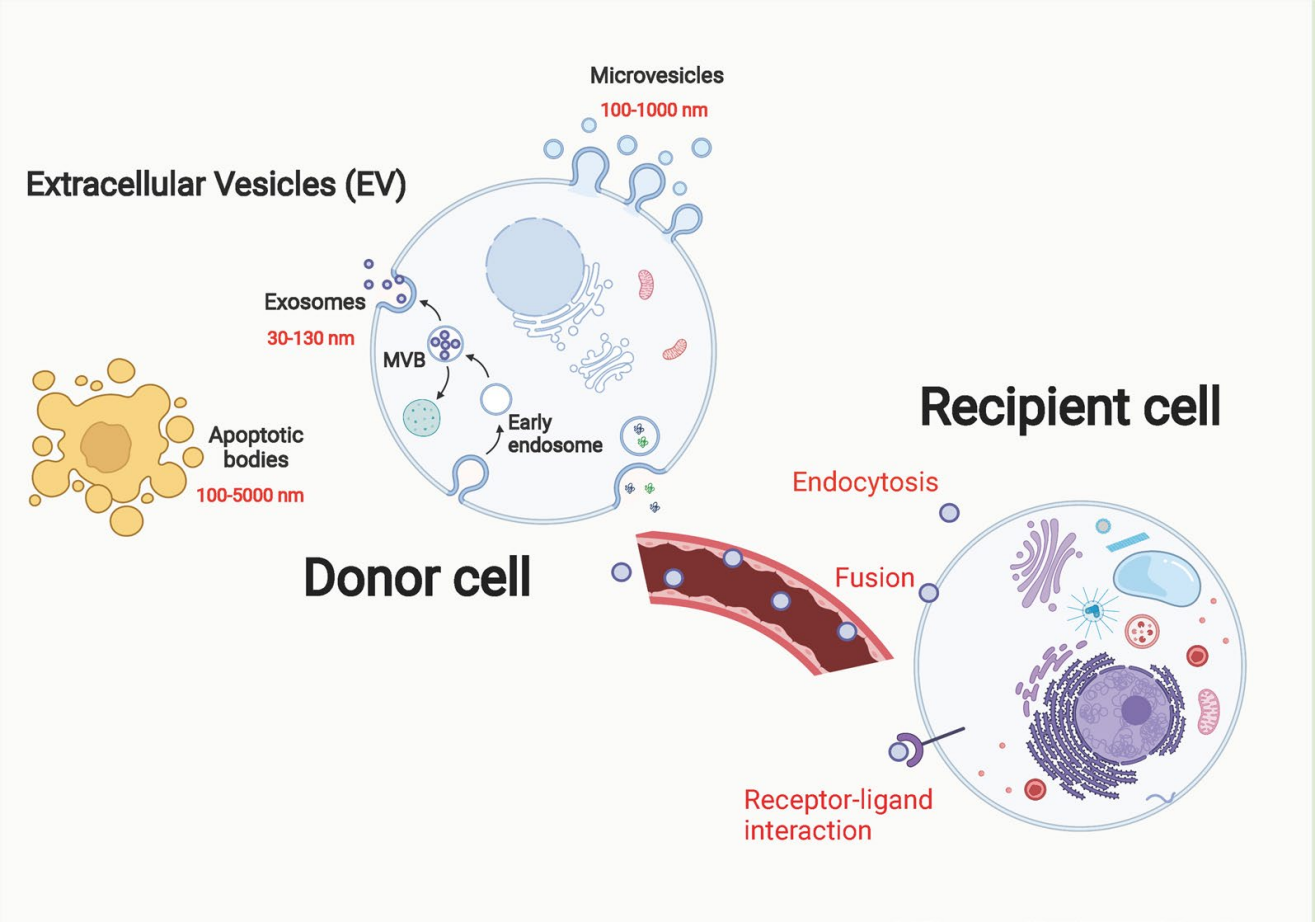
The main process of exosome biogenesis and release. Most cells in human body can release double-layer membrane-bound nanovesicles into the extracellular space. These membrane-derived vesicles can be divided into three types according to their size: small extracellular vesicles, microvesicles, and apoptotic bodies. Small extracellular vesicles originate from intracellular multivesicular bodies. Multivesicular bodies are vesicles with endocytosis. After fusion with the plasma membrane, a part is degraded by lysosomes, and the other part is released outside the cell to form small extracellular vesicles. Three main ways of information transmission exist between small extracellular vesicles and target cells. Exosome membrane proteins interact with target cell membrane proteins to activate intracellular signaling pathways. Small extracellular vesicles can transfer their own genetic material. The exosome membrane can be directly fused with the target cell membrane, and the genetic information carried in the exosome can be directly transferred to the recipient cell

SEVs are regarded as a class of extracellular vesicles (EVs) formed by endocytosis at the nanometer scale (30–130 nm in diameter), which are formed in the endosomal network and released to fuse with the plasma membrane [32–34]. SEVs and micro-vesicles (MVs) are both EVs, but their origins are different. MVs are generally 100–1000 nm in diameter and are usually membranous vesicles formed by the shedding of cytoplasm from the cell membrane [35, 36]. In contrast, SEVs originate from the endosome, also known as the multivesicular endosome (MVE) or multivesicular body (MVB) [37, 38]. In the maturation process, the intraluminal vesicles (ILVs) are released from the membrane and fused with the cell membrane to form the ILV, the exosome [39–41]. Upon arrival at the recipient cell, SEVs release their contents in the specific cell by binding to receptors, endocytosis, and fusion with the plasma membrane, thereby altering the cell’s physiological status and biological function [36, 42, 43] (Fig. 1).

**Fig. 2:**
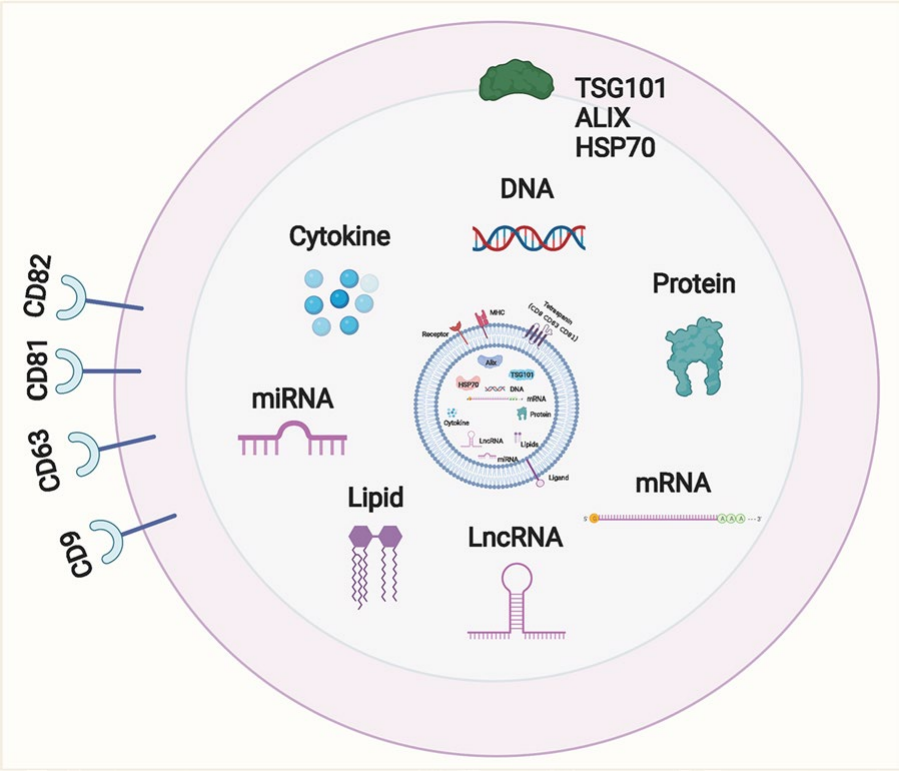
The contents of small extracellular vesicles. Almost all types of cells can secrete small extracellular vesicles, and small extracellular vesicles widely contain nucleic acids such as microRNA (miRNA), lncRNA, circRNA, mRNA, proteins, and lipids. Their surface markers mainly include CD63, CD81, CD9, ALG-2 Interacting protein X (Alix), tumor susceptibility gene 101 (TSG101), and heat shock protein 70 (HSP70)

SEVs primarily comprise nucleic acids, proteins, and lipids (Fig. 2). All SEVs commonly have proteins such as CD9, CD63, CD81, and CD82, which may serve as biomarkers and may also be related to the biological origin of SEVs [44, 45]. SEVs of different cellular origins express specific proteins, such as tumor susceptibility gene 101 protein (TSG101), ALG⁃2 interaction protein X (ALix), and heat shock protein 70 (HSP70), which are correlated to specific cellular functions [46, 47]. Additionally, SEVs contain a large number of nucleic acids, such as messenger RNA (mRNA), microRNAs (miRNAs), long non-coding RNAs (lncRNAs), and circular RNAs (circRNAs) [48–50]. These nucleic acids can fuse with target cells and act on the recipient cells to modulate gene expression and various signaling pathways in the recipient cells [51, 52]. SEVs contain lipids such as cholesterol, diglycerides, and phospholipids [53–56]. They are involved in the formation and maintenance of exosome morphology and serve as signaling molecules in the intercellular message communication process [57, 58]. These contents can be transported with body fluids to target cells to participate in angiogenesis, tumorigenesis, development, and metastasis [59–61].

TEXs have been reported to play a vital role in several aspects of tumorigenesis and progression. Recently, Wang et al. reported that Linc01091 encapsulated in TEXs could activate the ELF4/CDX2 axis by binding to miR-128-3p and facilitate the malignant progression of gastric cancer [62]. Moreover, it has been reported that inhibition of PD-L1 exosome release transforming growth factor-β (TGF-β) could work synergistically to promote the release of granzyme and interferon-γ to relieve the burden of tumor and depicts the regeneration of depleted T cells. This study established the role of TGF-β as a promoter of exosomal PD-L1 in breast cancer [63]. Moreover, transferring exogenous miR-25-5p carried in TEXs to anoikis-resistant hepatocellular carcinoma cells could significantly enhance cell motility and promote tumor self-implantation [64].

## The roles of TEXs in the initiation and development of glioma

TEXs were involved in glioma initiation and development by affecting TME, radiation therapy, chemoresistance, invasion and metastasis, angiogenesis, and tumor growth.

## The roles of TEXs in TME

**Fig. 3:**
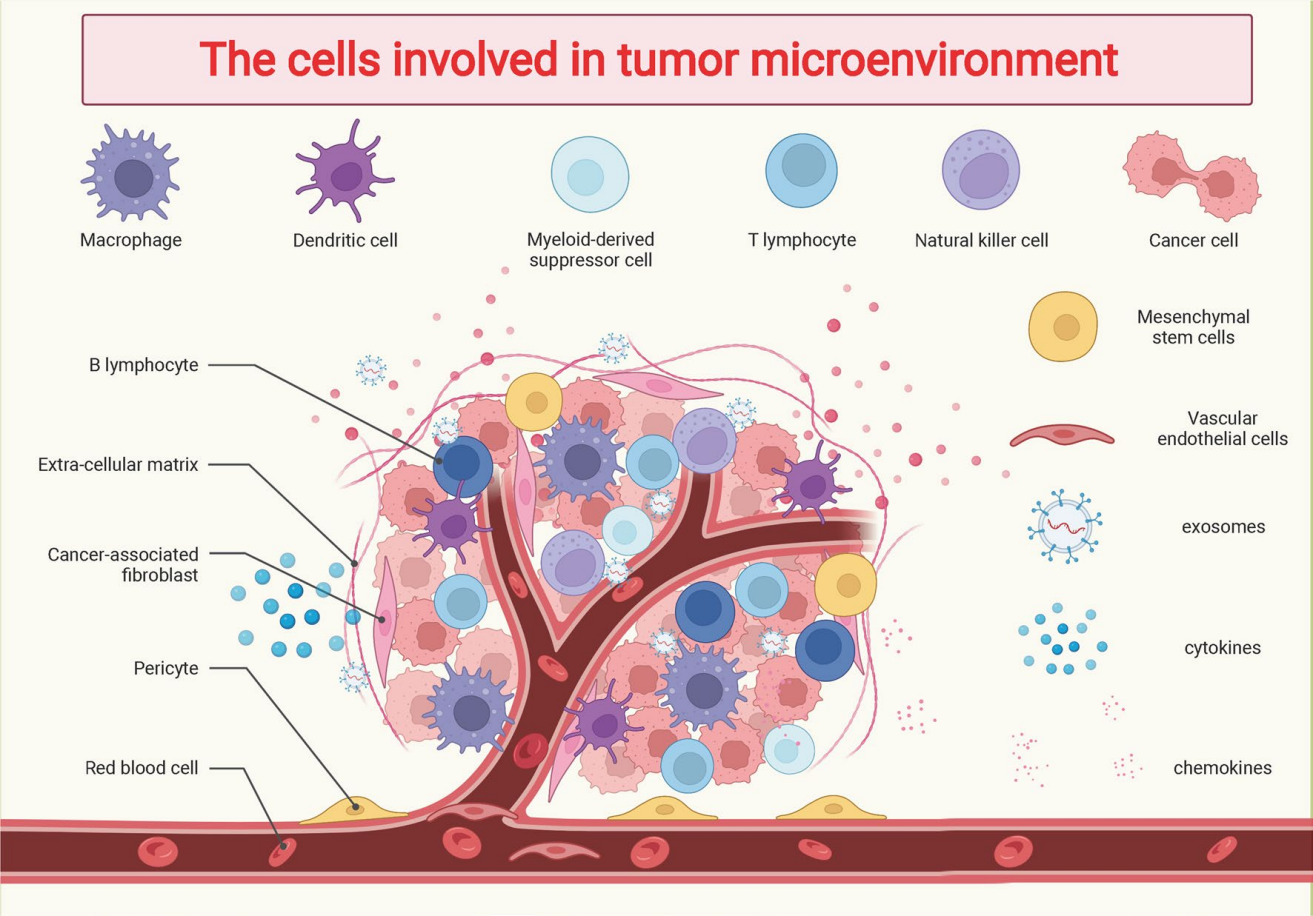
The cells involved in the tumor microenvironment. The tumor microenvironment is the growth environment of tumor cells, which is composed of tumor cells, immune cells, interstitial cells, extracellular matrix, and other factors, whose composition is more complex

The TME consists of tumor cells, immune cells, mesenchymal stem cells, vascular endothelial cells and non-cellular components such as ECM, cytokines, and chemokines, which can affect reversible changes in tumor cell phenotype and promote tumor cell metastasis and proliferation (Fig. 3). Many factors within the TME influence the interaction between tumor cells and immune cells, including the negative regulation of immune regulation by cytokines, polarization response of macrophages, and negative regulation of metabolic activity of T cells, which may suppress the killing effect of immune cells on tumor cells and allow tumor cells to undergo immune escape [65, 66].

The role of SEVs in intercellular communication, particularly during tumor development, has been extensively studied. SEVs deliver biologically active molecules such as proteins, mRNA, and miRNA to cells in the TME and thus, play an immunomodulatory role [67–69]. The role of SEVs in tumor immunity is double-edged, with both immunostimulatory and immunosuppressive effects; it acts mainly through regulating relevant immune cells in TME. SEVs can exert their biological effects by interacting with receptor cells through various mechanisms, including activation of cell surface receptors through receptor–ligand interactions [8, 70]. In addition, SEVs can deliver their contents to receptor cells via membrane fusion [71, 72], and receptor cells internalize SEVs through cytosolic drinking, phagocytosis, and endocytosis [17, 73].

Moreover, glioma cell-derived SEVs were reported to be involved in glioma development and progression by affecting TME (Table 1). CD133^+^ U87 glioblastoma cell-derived SEVs grown under hypoxic conditions are potent proliferation inducers of the tumor vascular system and glioma cell proliferation. CD133^+^ U87 glioblastoma cells may secrete exosome-derived miRNAs to promote angiogenic responses and glioma cell proliferation, which may be potential targetable drivers of hypoxia-dependent intercellular signaling upon tumorigenesis and progression [74]. Human astrocytoma U251 cell-derived SEVs induce tumorigenesis in human bone marrow mesenchymal stem cells (hBMSCs) by enhancing the cell proliferation, migration, and invasion; promoting the cell cycle; and activating glycolysis in hBMSCs [74]. Wang et al. reported that GBM could secrete multiple tumor-derived extracellular vesicles (TDEVs) with high immunosuppressive activity, thereby remotely suppressing the systemic immune system. The CD73^+^ TDEVs released by GBM cells could be taken up by T cells and inhibit cell cycle entry and clonal proliferation of T cells. Defects in exosome synthesis and CD73 expression remarkably repressed tumor growth in GBM-bearing mice and restored clonal proliferation of T cells in peripheral and central regions [75].

**Table 1:** Overview of TEXs cargos and their biological effects in TME

Donor cell	Exosomal cargos	Receiving cell	Biological effects	References
CD133 + U87	miRNAs	Vascular system cells	Promote angiogenic response	[74]
U251	/	hBMSCs	Promote proliferation, migration, invasion, cell cycle and glycolysis	[155]
GBM cells	CD73	T cells	Inhibit cell cycle and proliferation	[75]
Hypoxic glioma cells	miR-10a and miR-21	MDSCs	Target RORA and PTEN Induce MDSCs amplification and activation	[76]
GBM cells	miR-214-5p	primary microglia	Target CXCR5 Promote inflammatory response	[79]
GBM cells	miR-1246	M2 macrophage	Target TERF2IP/STAT3/NF-κB Induced M2 macrophage polarization	[80]
GBM cells	miR-155-3p	M2 macrophage	Target IL-6-pSTAT3-miR-155-3p-autophagy-pSTAT3 Induced M2 macrophage polarization	[81]
Glioma cells	circNEIL3	macrophage	Induce immunosuppressive properties	[82]

In addition, exosome-derived ncRNAs play a significant role in the malignant tension of gliomas. Normoxia-stimulated and hypoxia-stimulated glioma-derived SEVs (GDEs) were isolated. It was observed that hypoxia promotes the secretion of GDEs; myeloid-derived suppressor cells (MDSCs) can efficiently take up GDEs, and hypoxic glioma-derived SEVs (HGDEs) could significantly induce MDSCs compared with normoxic glioma-derived SEVs (N-GDEs) in vitro [76]. Hypoxia-induced expression of miR-10a and miR-21 in GDEs could mediate GDE-induced MDSC amplification and activation by targeting RORA and PTEN. Yang et al. reported that miR-214-5p expression was significantly higher in GBM and was strongly correlated to poorer clinical prognosis. Overexpression of miR-214-5p in cells markedly regulated cell proliferation and migration, which has been reported in various cancer cells [77, 78]. Mechanistic results demonstrated that GBM-derived exosomal miR-214-5p promotes inflammatory responses in primary microglia by targeting CXCR5, and thus, followed with lipopolysaccharide attack [79]. Qian et al. reported that HGDEs significantly induced M2 macrophage polarization compared with N-GDEs, thereby promoting glioma growth and metastasis. Furthermore, the next-generation sequencing results suggested that miR-1246 was enriched in the cerebrospinal fluid (CSF) of patients with GBM, and the expression was decreased after surgical resection. MiR-1246 could activate the STAT3 signaling pathway and repress NF-κB signaling-pathway-mediated H-GDE-induced M2-macrophage polarization via targeting TERF2IP and targeting miRNA-1246 might contribute to antitumor immunotherapy [80]. In addition, Xu et al. demonstrated that H-GDEs significantly promoted autophagy and M2-like macrophage polarization compared with N-GDEs, thereby promoting glioma growth and metastasis (possibly through an IL-6-pSTAT3-miR-155-3p-autophagy-pSTAT3 positive feedback loop), and in turn, its biological effects [81]. Pan et al. reported that circNEIL3 is packaged by hnRNPA2B1 into SEVs and delivered to infiltrating tumor-associated macrophages (TAM) via glioma cells, enabling them to acquire immunosuppressive properties via stabilizing IGF2BP3. This might, in turn, promote the malignant progression [82]. Mechanistically, circ-NEIL3 stabilizes the known oncogenic protein IGF2BP3 by blocking HECTD4-mediated ubiquitination.

## The roles of TEXs in radiation therapy

**Fig. 4:**
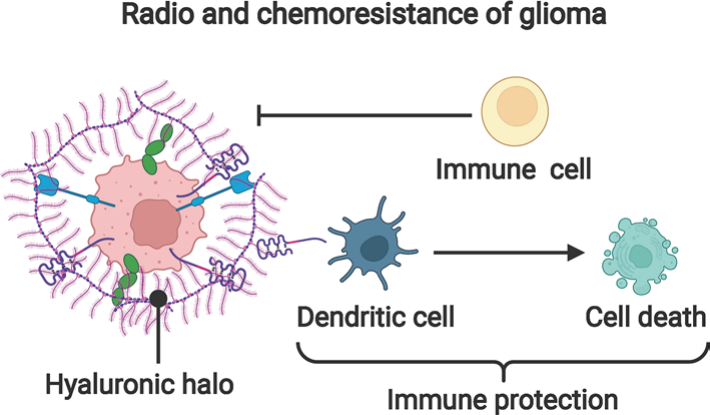
The involving participants mediating the radiation- and chemoresistance of glioma

Glioma-cell-derived SEVs have been reported to be involved in glioma development and progression by influencing radiation therapy (Table 2). The efficacy of radiation therapy as an important adjuvant therapy after surgical resection is well established for gliomas [1, 83] (Fig. 4). Radiation therapy can directly kill or inhibit the growth of residual tumors and prolong the survival time of patients. It has now become the standard treatment for high-grade glioma (HGG) [84].

**Table 2:** Overview of TEXs cargos and their biological effects in radiation therapy

Donor cell	Exosomal cargos	Receiving cell	Biological effects	References
Radiation tolerant GBM cells	AHIF	GBM cells	Regulate migration and angiogenesis related factors Promote cell survival, invasion and radiation tolerant	[85]
Hypoxic GBM cells	miR-301a	Normoxic GBM cells	Target TCEAL7/Wnt-β-cateenin Promote radiation tolerant	[86]
GBM cells	circ-METRN	GBM cells	Target miR-4709-3p/GRB14/PDGFRα signal Promote radiation tolerant	[103]

Dai et al. reported that antisense transcript of hypoxia-inducible factor 1α (AHIF) expression was remarkably increased in GBM tissues and GBM cells with radiation tolerance. Overexpression of AHIF in GBM cells significantly increased cell viability and invasiveness and inhibited apoptosis. Furthermore, SEVs from AHIF-overexpressing GBM cells significantly promoted cell viability, invasion, and radioresistance, possibly through regulating migration and angiogenesis-related factors [85]. Yue et al. reported that Exo-miR-301a, specifically secreted by hypoxic GBM cells, could be transferred to the normoxic cultured cells and further enhance cellular radio-resistance. Hypoxic Exo-miR-301a could directly target the *TCEAL7* gene and block the nuclear translocation of b-linked protein, thereby negatively regulating the Wnt/b-linked protein pathway [86]. Exo-miR-301a/TCEAL7 signaling axis may be a therapeutic target for radiation therapy resistance in patients with GBM.

## The roles of TEXs in chemoresistance

Tumor resistance is the insensitivity or reduced sensitivity of tumor cells to antitumor drugs that would normally kill tumor cells. The development of tumor resistance is a major cause of chemotherapy failure. Chemotherapy is an important treatment strategy for glioma (Fig. 4). The main mechanisms for developing drug resistance in glioma are heterogeneity, hypermutation, immune evasion, and selective splicing of tumor activation. Procarbazine, lomustine, and vincristine combination regimens and combination regimens of cytotoxic chemotherapeutic agents such as teniposide, etoposide, isocyclophosphamide, cisplatin, or carboplatin have been used occasionally, but these regimens have limited efficacy and greater cytotoxic effects [1, 87]. Temozolomide (TMZ) is a novel alkylating agent with good CNS permeability and it can reach nearly 100% bioavailability when administered orally. Compared with traditional cytotoxic chemotherapeutic agents, TMZ has mild side effects, is well tolerated, and is easy to administer [87, 88]. Currently, TMZ-based chemotherapy regimens have been widely used in the adjuvant treatment of high-grade gliomas, in the salvage treatment of recurrent gliomas, and low-grade gliomas with poor prognostic factors [89, 90].

Additionally, glioma-cell-derived SEVs could be involved in glioma development and progression by affecting chemoresistance (Table 3). A study characterized the SEVs of GBM cells with and without PTPRZ1-MET fusion (ZM fusion) and assessed the role of ZM exosome-mediated intercellular communication in the GBM microenvironment. The results revealed that ZM-derived SEVs significantly promoted GBM cell migration and invasion, neurosphere growth, and angiogenesis and promoted temozolomide resistance in GBM cells [91]. Expression levels of exosome-linked protein 43 (Cx43) were significantly higher in TMZ-resistant GBM cells compared with TMZ-sensitive cells. Exosome-derived Cx43 from TMZ-resistant GBM cells significantly enhanced cell migration and invasion and conferred TMZ resistance to receptor-sensitive cells, suggesting that Cx43 is expected to be a future therapeutic target for glioblastoma [92]. EV secreted by Glioma stem cells (GSC-EV) could be involved in radiation resistance and malignant progression of glioma. GSC-EV was reported to be taken up by human glioblastoma cell line LN229 and U118 receptor cells. These receptor cells survived radiation exposure and could efficiently form colonies, significantly enhancing cell migration and invasion. Mechanistic findings indicated that GSC-EV and its specifically wrapped miRNAs might induce phenotypic changes in the recipient cells by activating the PTEN/Akt pathway [93]. In addition, it was also shown that recombinant SEVs (R-EXO) from homologous glioma cells could carry TMZ and dihydrotanshinone (DHT), can reverse drug resistance, and enhance focal targeted drug delivery, defined as R-EXO-TMZ/DHT (R-EXO-T/D). R-EXO-T/D was reported to have multiple advantages, including good blood–brain barrier (BBB) penetration with nanometer size, tumor homing aggregation with homologous effects, and enhanced antitumor activity by overcoming TMZ resistance and triggering immune responses [94]. Macrophage movement inhibitory factor (MIF) expression is increased in SEVs of TMZ-resistant cells and can be transferred from TMZ-resistant cells to sensitive cells. Mechanistic studies have reported that exosome-derived MIF can enhance the sensitivity of drug-resistant glioma cells to TMZ via repressing the expression of metalloproteinase inhibitor 3 (TIMP3) and subsequently activating the PI3K/AKT signaling pathway [95].

**Table 3:** Overview of TEXs cargos and their biological effects in chemoresistance

Donor cell	Exosomal cargos	Receiving cell	Biological effects	References
Z-M fused GBM cells	/	GBM cells	Promote cell migration, invasion, neurosphere growth, angiogenesis and TMZ resistance	[91]
TMZ resistant GBM cells	Cx43	TMZ sensitive GBM cells	Promote cell migration, invasion and TMZ resistance	[92]
Glioma stem cells	miRNAs	Glioma cells	Target PTEN/Akt signal pathway Promote cell migration and invasion	[93]
TMZ resistant GBM cells	MIF	TMZ sensitive GBM cells	Target TIMP3/PI3K/AKT signal pathway Promote TMZ resistance	[95]
U87MG	miR-221	SHG-44	Target RELA/miR-221/DNM3 signal pathway Promote cell proliferation, migration and TMZ resistance	[96]
TMZ resistant GBM cells	miR-151a	TMZ sensitive GBM cells	Target miR-151a/XRCC4/DNA signal pathway Promote TMZ resistance	[97]
TMZ resistant GBM cells	miR-1238	TMZ sensitive GBM cells	Target miR-1238/CAV1/EGFR signal pathway Promote TMZ resistance	[156]
TMZ resistant GBM cells	SBF2-AS1	TMZ sensitive GBM cells	Target miR-151a-3p/XRCC4 Promote TMZ resistance	[99]
TMZ resistant GBM cells	miR-25-3p	TMZ sensitive GBM cells	Target miR-25-3p/FBXW7/c-myc/cyclin E Promote cell proliferation and TMZ resistance	[100]
GBM cells	lnc-TALC	TAM	Target ENO1/p38/MAPK Promote M2 polarization of microglia and TMZ resistance	[101]
TMZ resistant GBM cells	circNFIX	TMZ sensitive GBM cells	Target miR-132 Promote cell migration, invasion and TMZ resistance	[137]
TMZ resistant GBM cells	circ-HIPK3	TMZ sensitive GBM cells	Target miR-421/ZIC5 Promote cell growth, invasion and TMZ resistance	[102]
TMZ resistant GBM cells	circ-007208	TMZ sensitive GBM cells	Target miR-1252-5p/ALKBH5/NANOG Promote cell growth, invasion and TMZ resistance	[104]
TMZ resistant GBM cells	has_circ_0042003	TMZ sensitive GBM cells	Promote cell TMZ resistance	[105]

Recent studies have identified that exosome-derived non-coding RNAs play a significant role in chemoresistance in glioma. Yang et al. found that miR-221 expression was significantly increased in glioma tissues and SEVs, and inhibition of miR-221 expression in SHG-44 cells significantly inhibited cell proliferation, migration, and TMZ resistance; however, U87MG-derived SEVs produced protumorigenic effects [96]. u87MG-derived SEVs may contribute to glioma malignancy by regulating the RELA/. TMZ-resistant GBM cells may confer TMZ chemoresistance to the receptor by secreting SEVs into TMZ-sensitive cells and inhibiting the miR-151a/XRCC4/DNA repair signaling axis in the cells [97]. Furthermore, exosomal-miR-151a predicted chemotherapeutic response and is a potential therapeutic target for therapeutic GBM. In addition, miR-1238 expression levels were reported to be significantly higher in TMZ-resistant GBM cells and their SEVs than in TMZ-sensitive cells. TMZ-resistant GBM cells can promote their resistance by secreting miR-1238 into TMZ-sensitive GBM cells and directly targeting the CAV1/EGFR pathway [98]. SBF2-AS1 expression was upregulated in both TMZ-resistant GBM cells and tissues and overexpression of SBF2-AS1 in cells resulted in enhanced TMZ resistance. Mechanistic results revealed that TMZ-resistant GBM cells could reshape the TME and promote tumor chemoresistance by secreting exosome-derived SBF2-AS1 into TMZ-sensitive GBM cells and promoting XRCC4 expression through binding to miR-151a-3p [99]. Exosome-derived miR-25-3p was a TMZ-resistance-associated miRNA with remarkably higher expression in A172R cell SEVs and serum samples from patients with GBM treated with TMZ. Overexpression of miR-25-3p remarkably contributed to the proliferation and TMZ resistance of sensitive GBM cells. Mechanistic findings indicated that exosome-derived miR-25-3p might exert biological effects by promoting the expression of c-Myc and cyclin E through downregulation of FBXW7 [100].

Long non-coding RNA *Lnc*-TALC can be encapsulated into SEVs and delivered by GBM cells to TAM, thereby promoting M2 polarization in microglia. Lnc-TALC can bind to ENO1 and promote phosphorylation of p38 MAPK, thereby promoting C5/C5a fractionation. C5 can significantly promote the repair of TMZ-induced DNA damage, leading to chemoresistance. In conclusion, exosome-derived lnc-TALC can remodel the GBM microenvironment and reduce tumor sensitivity to TMZ chemotherapy [101].

It has been reported that exosome-derived circRNAs play a vital role in TMZ resistance. circNFIX expression is significantly increased in serum SEVs of TMZ-resistant patients and is strongly associated with poor prognosis. TMZ-resistant GBM-cell-derived exosome-derived circNFIX can enhance TMZ resistance by binding to miR-132 and directly interacting with miR-132. Moreover, exosome-derived circ-HIPK3 can promote TMZ-resistant GBM cell growth and TMZ resistance by modulating the miR-421/ZIC5 axis and participating in intercellular communication between GBM cells [102]. Low-dose radiation stimulates GBM cells to secrete circ-METRN-rich SEVs, and circ-METRN enhances glioblastoma progression and radioresistance by regulating the miR-4709-3p/GRB14/PDGFRα pathway [103]. Circ-0072083 in TMZ-resistant GBM tissues and cells Expression was significantly increased in both TMZ-resistant GBM tissues and cells. It promoted drug resistance by promoting IC_50_, proliferation, migration, invasion, and xenograft tumor growth of TMZ and inhibiting apoptosis. Mechanistic studies reported that circ-007208 could enhance ALKBH5-mediated demethylation and thus promote NANOG expression by binding to miR-1252-5p [104]. Acetyl heparinase was upregulated in TMZ-resistant GBM cells, and overexpression of acetylheparinase significantly increased the resistance of U87 cells to TMZ. In addition, acetyl heparinase promoted exosome secretion by GBM cells and mediated the transfer of exosome-derived has_circ_0042003 from TMZ-resistant glioma cells to drug-sensitive cells [105].

## The roles of TEXs in invasion and metastasis

**Fig. 5:**
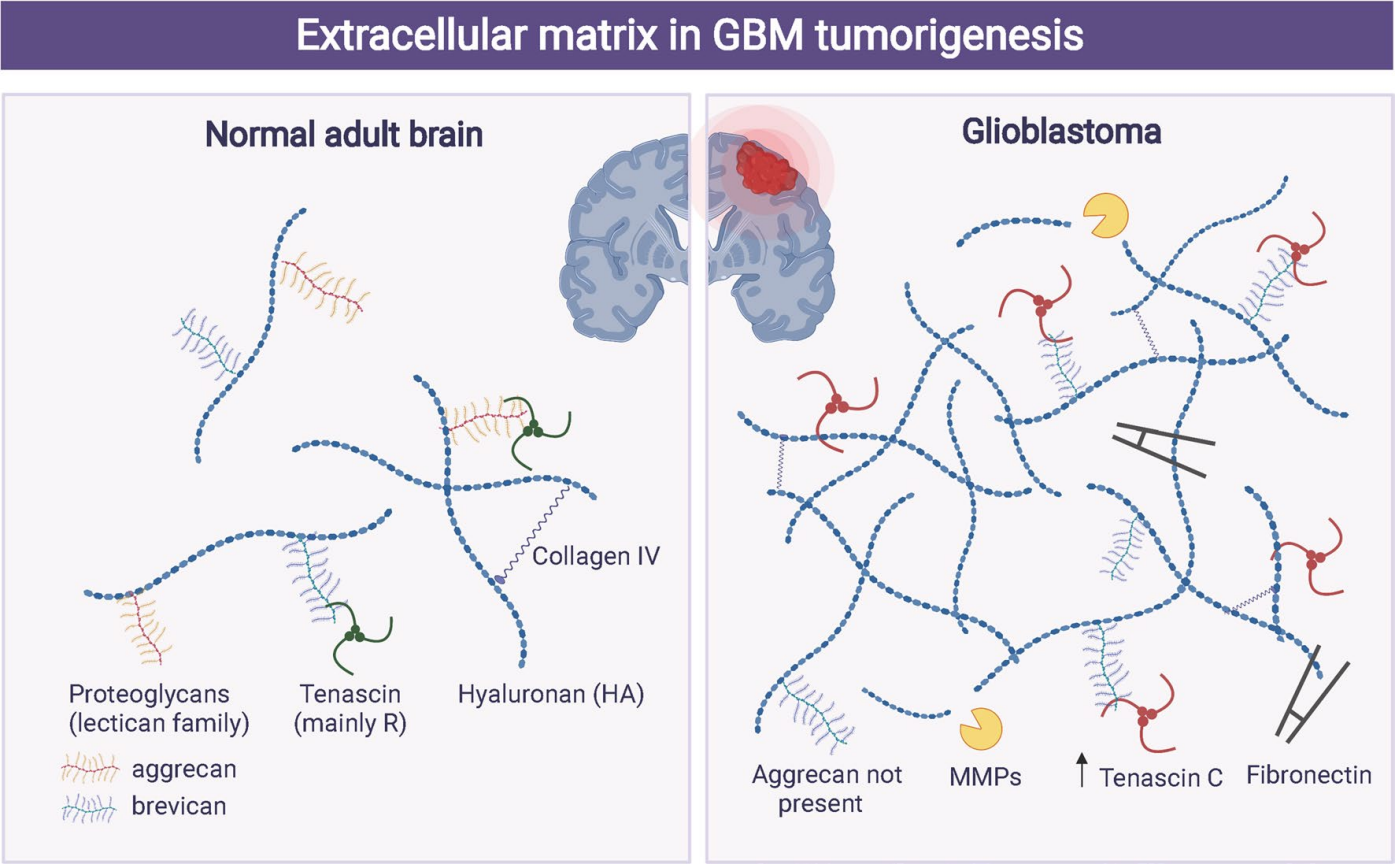
The extracellular matrix in GBM tumorigenesis. The features of extracellular matrix in normal brain are low overall density and stiffness and absence of neurodevelopmental proteins. The features of extracellular matrix in GBM are increased overall density and stiffness, presence of oncofetal protein isoforms, and promotion of invasion and angiogenesis

Invasion and metastasis are the important reasons underlying poor tumor prognosis, and their occurrence involves several factors such as weakened adhesion between tumor cells and degradation of extracellular matrix, which can promote tumor metastasis [106, 107]. Extracellular matrix (ECM) acts as a natural barrier to tumor invasion and metastasis, and thus effectively prevents tumor metastasis [108, 109] (Fig. 5). During the establishment of the premetastatic niche, ECM changes through reorganization or a new ECM is deposited. Many factors contribute to this change, including solubility factors, immune cells, and exosomes, which is to create a permissive seeding and growth environment of circulating cancer cells [110]. Tumor-derived exosomes regulate ECM, and exosome-induced fibronectin deposition has been reported in both liver and lung premetastatic niches. In the liver, PaC exosomes carrying macrophage migration inhibitory factor (MIF) promote the secretion of TGF-b by Kupffer cells, thereby inducing stellate cells to produce fibronectin [111]. Exosomal small nuclear RNA enhances the expression of metalloproteinase-9 (MMP-9) and fibronectin in the lung premetastasis niche, thereby promoting the recruitment of neutrophils [112]. Moreover, non-small cell lung cancer (NSCLC) cells modulated the expression of podocalyxin in exosomes, which in turn impacted integrin trafficking in fibroblasts and created a supportive microenvironment for tumor cell migration and invasion by introducing tumor-promoting ECM components [113]. All these aforementioned studies highlighted the role of SEVs in modulating ECM.

Glioma-cell-derived SEVs were reported to be involved in glioma development and progression via influencing tumor invasion and metastasis (Table 4). MiR-148a levels in circulating SEVs were significantly higher in the serum of patients with GBM than in healthy volunteers. Inhibition of miR-148a expression in glioma cells significantly inhibited cancer cell proliferation and metastasis. The results of mechanistic experiments suggested that exosome-mediated miR-148a may promote cancer cell proliferation and metastasis by targeting cell adhesion molecule 1 (CADM1) to activate the STAT3 pathway [114]. Glioma-cell-derived SEVs can deliver lnc-ATB to astrocytes and activate astrocytes by suppressing miR-204-3p expression in an Ago2-dependent manner. Surprisingly, lnc-ATB-activated astrocytes could, in turn, promote glioma cell migration and invasion, indicating that lnc-ATB might contribute to regulating the glioma microenvironment through exosomal forms [115]. MiR-375 expression was significantly downregulated in gliomas; it could inhibit glioma growth by repressing the CTGF-epidermal growth factor receptor (EGFR) signaling pathway, thus inhibiting glioma proliferation, migration, and invasion. In addition, exosome-derived miR-375 was significantly downregulated in peripheral blood samples from patients with glioma and was strongly associated with patient prognosis. Exosome-derived miR-375 inhibits glioma cell proliferation and invasion through sustained inhibition of the CTGF-EGFR oncogenic pathway [116]. miR-1246 and miR-10b-5p are significantly upregulated in H-GDEs and can be delivered to normoxic glioma cells, promoting the migration and invasion of normoxia cells in vitro and in vivo. Mechanistic studies have reported that miR-1246 and miR-10b-5p can induce glioma migration and invasion by directly targeting FRK and TFAP2A [117]. Glioma cells secrete SEVs that significantly promote the malignant progression of gliomas, and exosome-derived circ-0001445 can be taken up by glioma cells. Circ-0001445 entering glioma cells can act as a sponge for miR-127-5p and upregulate the expression of sorting linker protein 5 (SNX5), which promotes glioma migration and invasion [118]. Exosome-derived circZNF652 is significantly up-day expressed in glioma cells and can be taken up by other glioma cells. The entry of circ-0001445 into glioma cells promotes glioma migration and invasion by regulating the miR-486-5p/SERPINE1 signaling axis and the epithelial–mesenchymal transition process [93].

**Table 4:** Overview of TEXs cargos and their biological effects in invasion and metastasis

Donor cell	Exosomal cargos	Receiving cell	Biological effects	References
Glioma cells	miR-148a	Glioma cells	Target CADM1/STAT3 signal Promote cell proliferation and metastasis	[114]
Glioma cells	lnc-ATB	astrocytes	Target Ago2/miR-204-3p signal Induce astrocytes	[115]
Glioma cells	miR-375	Glioma cells	Target CTGF-EGFR signal Inhibit cell proliferation and invasion	[116]
Hypoxia glioma cells	miR-1246 and miR-10b-5p	Normoxia glioma cells	Target FRK and TFAP2A Promote cell migration and invasion	[117]
Glioma cells	circ-0001445	Glioma cells	Target miR-127-5p/SNX5 Promote cell migration and invasion	[118]
Glioma cells	circZNF652	Glioma cells	Target miR-486-5p/SERPINE1 Promote cell migration, invasion and EMT	[93]

## The roles of TEXs in angiogenesis

**Fig. 6:**
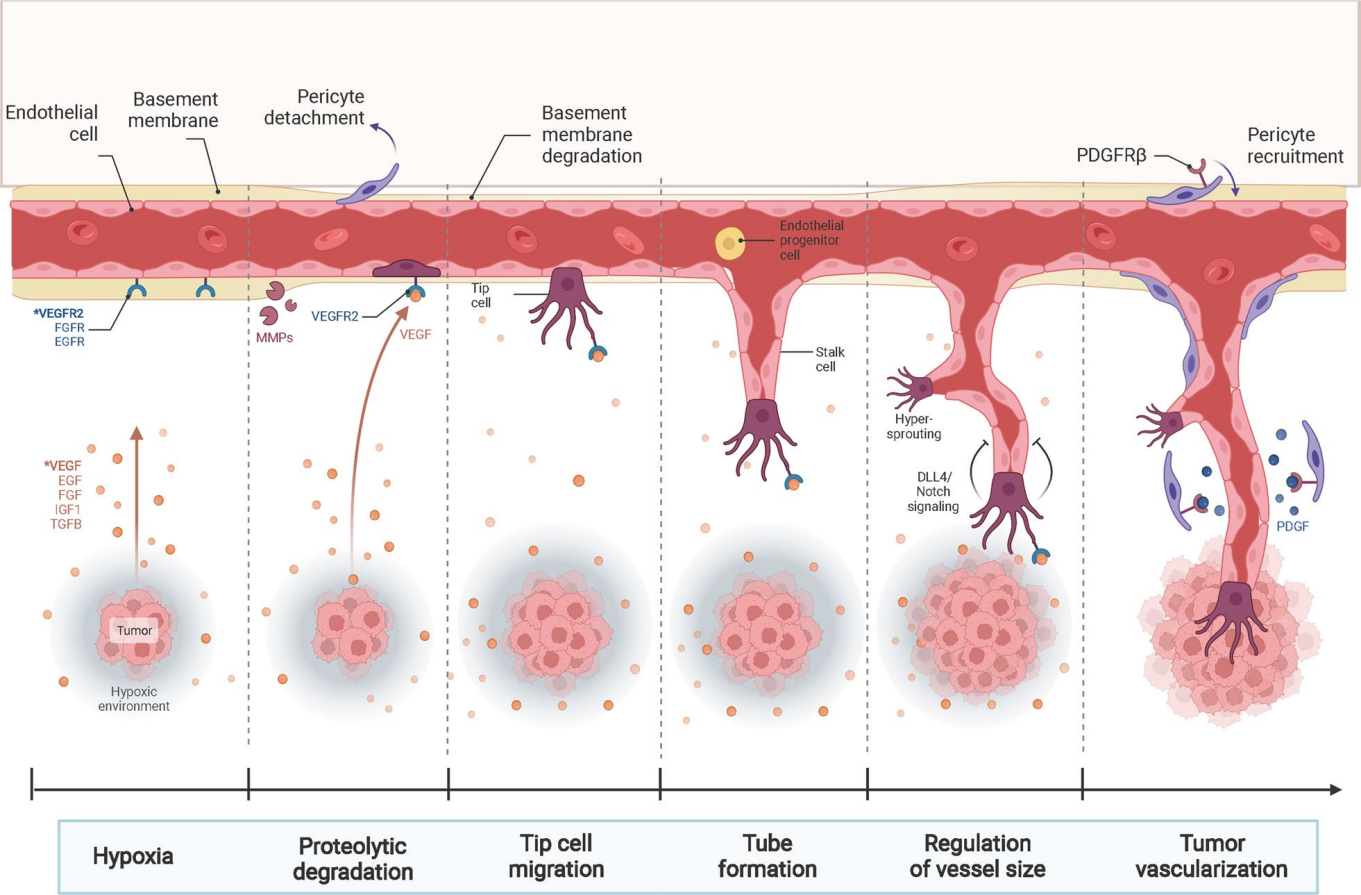
The features of tumor angiogenesis. Tumor angiogenesis is a complex process of interaction between tumor cells and endothelial cells. The main process is as follows: endothelial cells are stimulated by proangiogenic growth factors secreted by tumor cells. After degrading, endothelial cells proliferate under the action of various chemokines, form lumen with pericytes under the action of adhesion molecules, and generate new tumor blood vessels after maturation and stabilization. Tumor angiogenesis is an important basis for tumor growth, invasion, and metastasis. This process is regulated by various angiogenic growth-promoting or inhibitory factors and signaling pathways

Tumor angiogenesis plays a vital role in tumorigenesis and development. Angiogenesis refers to developing new blood vessels from existing capillaries or postcapillary veins [119, 120]. Generally, the whole process of angiogenesis occured in tissues is coordinated by angiogenic and vasopressor factors [121, 122]. Under external factors, internal genetic mutations, and tumorigenesis, the angiogenic factors are overactivated, while the vasopressor is suppressed. This kind of imbalance can activate the angiogenic system, resulting in excessive tissue angiogenesis [123, 124]. The rapid increase in blood vessels to meet the needs of tumor growth can lead to rapid tumor growth and increase the probability of tumor cell spread and metastasis (Fig. 6).

Glioma-cell-derived SEVs were reported to be involved in glioma development and progression by affecting tumor angiogenesis (Table 5). A specific 120-kDa vascular endothelial growth factor (VEGF) isoform, namely, VEGF-C, was reported to be present in GBM-derived SEVs, which binds to VEGF receptor 2 (VEGFR2). Further, it was reported that VEGF-C in GBM-derived SEVs exhibited a strong stimulatory effect on tafazzin (TAZ) expression in endothelial cells via suppressing the Hippo signaling pathway, which ultimately stimulates endothelial cell viability, migration, and tubularization [125]. Linc-CCAT2 is overexpressed in glioma tissues and significantly leads to the malignant progression of gliomas. Further studies have reported that linc-CCAT2-rich glioma-cell-derived SEVs can be taken up by HUVEC cells and can increase the expression level of linc-CCAT2 in HUVEC cells in turn promoting HUVEC migration, proliferation, tubular formation in vitro, and small artery formation in vivo and inhibits hypoxia-induced HUVEC cell apoptosis. Mechanistic studies have reported that linc-CCAT2 can upregulate the expression of VEGFA and TGFβ, promote the expression of Bcl-2, and inhibit the expression of Bax and caspase-3 in HUVEC cells [126].

**Table 5:** Overview of TEXs cargos and their biological effects in angiogenesis

Donor cell	Exosomal cargos	Receiving cell	Biological effects	References
GBM cells	VEGF-C	Endothelial cells	Target Hippo/TAZ signal Stimulate cell viability, migration, and tubulization	[125]
GBM cells	linc-CCAT2	HUVECs	Target VEGFA/TGF/Bcl-2/Bax/caspase-3 signal	[126]
			Promote cell migration, proliferation, tubular formation structure formation in vitro and arteriole in vivo	
Glioma stem cells	miR-21	Endothelial cells	Target miR-21/VEGF signal	[127]
GBM cells	VEGF-A	Endothelial cells	Promote angiogenesis and vascular permeability	[128]
Glioma stem cells	miR-26a	HUVECs	Target PTEN/PI3K/Akt Stimulate cell viability, migration, and tubulization	[129]
GBM cells	miR-182-5p	HUVECs	Target KLF2/KLF4 Stimulate cell proliferation and tubulization	[130]
GBM cells	miR-148a-3p	HUVECs	Target ERRFI1/EGFR/MAPK Stimulate cell proliferation and tubulization	[131]
Glioma stem cells	miR-944	HUVECs	Target VEGFC/AKT/ERK Inhibit cell viability, migration, and tubulization	[132]
GBM cells	circGLIS3	Endothelial cells	Target Ezrin T567 Stimulate tubulization	[133]

Studies have reported that glioma stem cells may contribute to the prognosis of glioblastoma by mediating cellular communication, mainly through the secretion of exosome-derived miRNAs. miR-21 expression is increased in glioblastoma, and it upregulates VEGF expression. Mechanistic experiments revealed that GSC-EXs could promote the angiogenic capacity of endothelial cells (ECs) through miR-21/VEGF signaling [127]. Proangiogenic propermeability factor (VEGF-A) is enriched in SEVs from the cells originating from patients with glioma and contributes to the increased permeability and angiogenic potential of human brain endothelial cells in vitro. Inhibition of VEGF-A expression significantly inhibited the increase in extracellular vesicle-mediated permeability and angiogenesis in vitro. Therefore, targeting EVs released from gliomas may be a therapeutic tool to inhibit tumor-induced angiogenesis and vascular permeability in GBM [128]. miR-26a is enriched in GSCs SEVs and promotes proliferation, migration, tube formation, and angiogenesis in HBMEC via targeting PTEN to activate PI3K/Akt signaling pathway [129]. Circulating miR-182-5p levels are elevated in serum and cerebrospinal fluid samples from patients with glioma, and its expression level is negatively correlated with prognosis. Under hypoxic conditions, miR-182-5p expression was significantly increased in the SEVs of GBM cells, which directly inhibited KLF2 and KLF4 and led to the accumulation of VEGFR, thereby promoting HUVEC cell proliferation and tumor angiogenesis. In addition, exosome-mediated miR-182-5p inhibits tight junction-related proteins (such as ZO-1, occludin, and claudin-5), thereby enhancing vascular permeability and transendothelial migration of tumors [130]. miR-148a-3p is enriched in glioma-cell-derived SEVs and can be transferred to HUVEC cells in an exosome-mediated form and promotes its proliferation. HUVEC and promote their proliferation and angiogenesis. Mechanistic findings suggested that miR-148a-3p activates the epidermal growth factor receptor (EGFR)/mitogen-activated protein kinase (MAPK) signaling pathway by inhibiting ERRFI1 expression [131]. It was reported that miR-944 levels were significantly lower in high-grade gliomas (HGGs) than in low-grade gliomas (LGGs), and overall survival was significantly lower in patients with glioma with low miR-944 expression than in patients with glioma with high miR-944 expression. In addition, GSC-derived exosome-derived miR-944 was delivered to HUVEC cells and significantly reduced cell proliferation, migration, and test tube formation in vitro. Mechanistic findings revealed that miR-944 significantly reduced VEGFC levels and inhibited activation of the AKT/ERK signaling pathway [132]. circGLIS3 expression was significantly increased in HGGs, and it promoted migration and invasion of glioma cells, which exhibited an aggressive profile in hormonal mice. Mechanistic findings suggested that circGLIS3 promotes the phosphorylation level of Ezrin T567. In addition, gliomas can secrete circGLIS3 into endothelial cells via SEVs and induce endothelial cell angiogenesis, thereby promoting glioma invasion and angiogenesis[133].

## The roles of TEXs in tumor growth

Glioma-cell-derived SEVs have been reported to be involved in glioma development and progression by affecting tumor growth (Table 6). It was reported that treating non-GSC glioma cells with GSC SEVs significantly enhanced cell proliferation, neurosphere formation, invasiveness, and tumorigenicity. Further studies revealed that the Notch1 signaling pathway was activated in GSC and highly enriched in GSC SEVs. GSC could deliver SEVs to nonGSC glioma cells and increase Notch1 expression, which in turn mediated the dedifferentiation of nonGSC glioma cells into GSC and enhanced the stemness and tumorigenicity of nonGSC glioma cells [134]. Linc01060 was upregulated in gliomas and significantly correlated with tumor grade and poor clinical prognosis. Linc01060 expression was significantly increased in hypoxic GSC (H-GSC), which promoted malignant proliferation of cells by transferring SEVs into glioma cells and leading to significantly higher Linc01060 expression in cells. Mechanistic findings suggested that Linc01060 promotes nuclear translocation of MZF1 and facilitates MZF1-mediated c-Myc transcriptional activity, whereas c-Myc enhances the accumulation of hypoxia-inducible factor-1α (HIF1a) at post-transcriptional levels. HIF1a binds to the hormone-responsive element of the Linc01060 promoter and upregulates Linc01060 gene transcription. Overall, inhibiting Linc01060-containing SEVs or targeting the Linc01060/MZF1/c-Myc/HIF1a axis may be an effective therapeutic strategy for glioma [135]. ROR1-AS1 is upregulated in glioma tissues, and high expression of ROR1-AS1 predicts poor prognosis in patients with glioma. ROR1-AS1 can be packaged into the SEVs of glioma cells and can significantly promote cell growth and metastasis. Mechanistic findings suggested that ROR1-AS1 acts as a sponge for miR-4686 and inhibits its expression. Tumor-cell-derived Exo-ROR1-AS1 may be a target for clinical treatment of glioma [136].

**Table 6:** Overview of TEXs cargos and their biological effects in tumor growth

Donor cell	Exosomal cargos	Receiving cell	Biological effects	References
Glioma stem cells	Notch1	Glioma cells	Promote cell stemness and tumorigenicity	[134]
Glioma stem cells	linc01060	Glioma cells	Target MZF1/c-Myc/HIF1a Promote cell growth	[135]
Glioma cells	ROR1-AS1	Glioma cells	Target miR-4686 Promote tumor growth	[136]

## TEXs serve as diagnostic and prognostic biomarkers in glioma

The search for a specific and nontoxic tumor marker has become increasingly urgent because of the late detection of gliomas, which are often not very effectively cured. In recent years, it has been reported that SEVs promise new tumor markers because they are widely distributed in eukaryotic cells, and the contents of SEVs secreted by various types of tumor cells can be helpful in the diagnosis of various tumors.

Patients with recurrent GBM with higher serum exosomal SBF2-AS1 levels had a worse prognosis, forecasting a poor response to TMZ treatment [99]. Exosomal circNFIX is biologically important in the early diagnosis of patients with recurrent GBM and prognostic assessment [137]. Detection of circ-METRN expression levels in serum SEVs of patients with glioma early in radiation therapy not only helps to predict radioresistance and prognosis but also assists in the early detection of glioblastoma recurrence by MRI [103]. circ_0072083 expression levels were significantly elevated in serum SEVs of drug-resistant patients with glioma and predicted a lower overall survival of patients [104]. Glioma can be diagnosed early by measuring the expression level of circulating exosomal miR-148a [114]. The prognosis of patients with glioma can be assessed by measuring the expression level of circulating exosomal miR-375 [116].

## Future prospections and conclusions

TEXs are rich in protein, miRNA, mRNA, DNA, and lipid contents and play an essential role in the early diagnosis, development, and treatment of tumors [138–140]. However, because of the differences in experimental materials and research methods, the detection of single exosome contents is prone to false-positive results. Therefore, there is still a long way to go for the clinical application and promotion of TEXs. To improve the accuracy of exosome content detection technology, we can focus on developing multiple exosome content assays in the future so that exosome-related developments can be better applied for the early diagnosis, treatment, and prognosis of tumors [141, 142].

Tumor-cell-derived SEVs are released into the extracellular space to bind to receptors on target cells through the humoral circulation, and transport characteristic proteins and nucleic acids. They are essential in promoting tumor invasion and metastasis [101, 143, 144]. Therefore, clarifying the function of SEVs is vital in understanding their role in the TME. With the in-depth study of SEVs, we have gained a deep understanding of the interactions and mechanisms between tumor cells, which provides a new theoretical basis for diagnosing and treating tumors. For a long time, studies on SEVs have focused on developing specific targeted antitumor vaccines. Their intrinsic mechanisms and their immune-enhancing or tolerogenic nature should be explored. Because SEVs are widely distributed in body fluids and have a long half-life, they may be suitable drug carriers. However, the feasibility of SEVs as drug carriers needs to be investigated. In conclusion, with the deepening of the understanding of SEVs and the clarification of tumor detection, diagnosis, and treatment methods based on exosome research, new antitumor drugs and clinical tumor interventions are expected to be developed.

Under both physiological and pathological conditions, SEVs mediate the exchange of information between cells and their surroundings [145–149]. TEXs have emerged as a major communication mechanism between tumor cells and TMEs and have a vital role in tumor progression and metastasis [11, 150–154]. The application of TEXs in the diagnosis and treatment of glioma is still in the nascent stages. Follow-up studies should focus on the biogenesis and secretion of TEXs, their interaction with target cells, and the role of exosomal components of TEXs, which are expected to improve the application of drug therapy and increase the survival rate of patients with glioma. However, still, some issues are to be resolved. For example, the sensitivity and specificity of TEXs in the early diagnosis and prognostic assessment of glioma still need to be improved. The acquisition of high-purity SEVs remains a challenge due to technical limitations and high costs, and the presence of impurity proteins in the extracted SEVs may affect the efficiency of the assay. The quantification, purification, and preservation of SEVs have not been standardized SEVs as tumor micro. The specific mechanism of SEVs as an important component of the TME in glioma evolution is still unclear. The adverse effects of SEVs in targeted therapy cannot be fully determined. These problems limit the application of TEXs in treating glioma.

This article reviewed the multifaceted nature of TEXs and their biological role in glioma genesis and development. In the future, the clinical application of TEXs will provide a new path for the treatment of glioma.

## Data Availability

The data in the current study are available from the corresponding authors on reasonable request.

## Abbreviations

TEXs: Tumor-derived exosomes
GBM: Glioblastoma multiforme
EVs: Extracellular vesicles
MVs: Micro-vesicles
MVE: Multivesicular endosome
MVB: Multivesicular body
ILV: Intraluminal vesicle
TSG101: Tumor susceptibility gene 101 protein
Alix: ALG-2 interaction protein X
HSP70: Heat shock protein 70
mRNA: Messenger RNA
miRNA: MicroRNA
lncRNAs: Long non-coding RNA
circRNA: Circular RNA
TME: Tumor microenvironment
hBMSCs: Human bone marrow mesenchymal stem cells
TDEVs: Tumor-derived extracellular vesicles
GDEs: Glioma-derived exosomes
HGDEs: Hypoxic glioma-derived exosomes
N-GDEs: Normoxic glioma-derived exosomes
CSF: Cerebrospinal fluid
TAM: Tumor-associated macrophages
AHIF: Hypoxia inducible factor 1α
TMZ: Temozolomide
Cx43: Exosome-linked protein 43
BBB: Blood–brain barrier
MIF: Macrophage movement inhibitory factor
TIMP3: Metalloproteinase inhibitor 3
ECM: Extracellular matrix
CADM1: Cell adhesion molecule 1
Ago2: Argonaute 2
EGFR: Epidermal growth factor receptor
SNX5: Sorting linker protein 5
VEGF: Vascular endothelial growth factor
ECs: Endothelial cells
HGGs: High-grade gliomas
LGGs: Low-grade gliomas
